# RNA-Sequencing Analysis of 5' Capped RNAs Identifies Many New Differentially Expressed Genes in Acute Hepatitis C Virus Infection

**DOI:** 10.3390/v4040581

**Published:** 2012-04-16

**Authors:** Neven Papic, Christopher I. Maxwell, Don A. Delker, Shuanghu Liu, Bret S. E. Heale, Curt H. Hagedorn

**Affiliations:** 1 Department of Medicine, University of Utah, 30 N 1900 E #3C310, Salt Lake City, UT 84132, USA; Email: neven.papic@hsc.utah.edu (N.P.); christopher.maxwell@hsc.utah.edu (C.I.M.); don.delker@hsc.utah.edu (D.A.D.); shuanghu.liu@hsc.utah.edu (S.L.); bret.heale@hsc.utah.edu (B.S.E.H.); 2 Huntsman Cancer Institute, University of Utah, 30 N 1900 E #3C310, Salt Lake City, UT 84132, USA; 3 Department of Experimental Pathology, University of Utah, 30 N 1900 E #3C310, Salt Lake City, UT 84132, USA

**Keywords:** HCV, JFH-1, RNA-seq, gene expression, next generation sequencing, 5' cap, Huh 7.5, FUT1, KLHDC7B

## Abstract

We describe the first report of RNA sequencing of 5' capped (Pol II) RNAs isolated from acutely hepatitis C virus (HCV) infected Huh 7.5 cells that provides a general approach to identifying differentially expressed annotated and unannotated genes that participate in viral-host interactions. We identified 100, 684, and 1,844 significantly differentially expressed annotated genes in acutely infected proliferative Huh 7.5 cells at 6, 48, and 72 hours, respectively (fold change ≥ 1.5 and Bonferroni adjusted *p*-values < 0.05). Most of the differentially expressed genes (>80%) and biological pathways (such as adipocytokine, Notch, Hedgehog and NOD-like receptor signaling) were not identified by previous gene array studies. These genes are critical components of host immune, inflammatory and oncogenic pathways and provide new information regarding changes that may benefit the virus or mediate HCV induced pathology. RNAi knockdown studies of newly identified highly upregulated *FUT1* and *KLHDC7B* genes provide evidence that their gene products regulate and facilitate HCV replication in hepatocytes. Our approach also identified novel Pol II unannotated transcripts that were upregulated. Results further identify new pathways that regulate HCV replication in hepatocytes and suggest that our approach will have general applications in studying viral-host interactions in model systems and clinical biospecimens.

## 1. Introduction

Chronic hepatitis C virus (HCV) infection is a major cause of chronic liver disease and hepatocellular carcinoma (HCC) worldwide [1,2,3]. The mainstay of treatment for chronic hepatitis C has been ribavirin and pegylated interferon-α that generally result in a 55% sustained viral response (clearance) in previously untreated patients [4,5,6]. While the addition of recently clinically tested HCV protease inhibitors and other direct-acting antiviral drugs have markedly improve the overall response to therapy in many patients, a significant proportion of patients with chronic hepatitis C remain unable to be treated with these regimens [4,5,6]. This includes patients who are intolerant of combinations of new antivirals or represent special cases, such as post liver transplant patients [4,5,6]. In addition, developing vaccines to HCV have been challenging [7]. A better understanding of HCV-host interactions is likely to have applications in developing better models of HCV infection and replication, improved antiviral therapies and vaccine strategies. 

To better understand early events in HCV infection, gene expression studies of *in vitro* acutely infected human cells have been performed. These studies have identified alterations in biologic pathways including TGF-beta signaling, apoptosis, cellular metabolism, and oxidative stress [8,9,10]. Interestingly, interferon signaling and RIG-I signaling pathways were not represented in previous gene expression studies and it was thought that Huh 7.5 hepatoma cells were unable to produce a RIG-I response [8].

The goal of this study was to perform the most inclusive analysis of changes in Pol II RNAs, including mRNAs with short 3' pol(A) ends, primary miRNA transcripts and long noncoding RNAs that lack 3' poly(A) ends, during acute HCV infection of Huh 7.5 cells [11,12,13]. We have reported previously that the analysis of 5' capped RNA, rather than poly(A) selected RNA, provides a more sensitive means of detecting differentially expressed genes in HCV infected biospecimens [14]. Our approach purifies Pol II transcripts by binding their 5' caps with a high-affinity variant of the RNA cap binding protein (eIF4E_K119A_), enabling us to isolate and study transcripts with short or missing 3' poly(A) ends [12,13,15,16,17]. This approach also identified a number of unannotated non-coding RNAs on chromosome 14 that are differentially expressed during chronic HCV infection [14]. These and other non-coding RNAs may be important in the host response to HCV infection or HCV replication as reported for miR122 [18,19,20].

In this study we used next generation RNA sequencing analysis of 5' capped RNA isolated from Huh 7.5 cells acutely infected *in vitro* with HCV to better characterize the early changes in hepatocyte gene expression during HCV infection. These analyses provide new insights into HCV-induced changes in host gene expression, including identifying genes that might be important in viral replication, virus assembly, and hepatocellular carcinoma development in chronically infected HCV patients. Our results include RNAi knockdown studies of genes that are markedly upregulated during acute HCV infection and findings that their expression facilitates viral replication *in vitro*. 

## 2. Results

### 2.1. HCV JFH-1 Infection of Huh 7.5 Cells

Triplicate cultures of Huh 7.5 cells were infected with HCV JFH-1 for 2 hours with an MOI of 0.5 virions/cell. The acute infection of Huh 7.5 cells produced infectious virus and approximately 40, 80, and 95 percent of cells were infected at 6, 48 and 72 hours, respectively, as measured by NS5A immunostaining. Control cells were mock infected at each time point. Pol II RNA transcripts (5' capped RNA) were isolated as previously described from total RNA recovered from infected and control cells after 6, 48 and 72 hours ([14], Experimental Procedures). 

### 2.2. RNA Sequencing (RNA-seq) Analysis of Annotated Gene Transcripts

**Figure 1:**
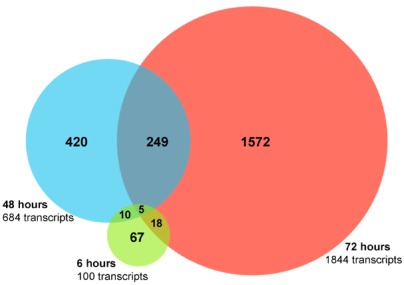
Differentially expressed annotated genes after acute hepatitis C virus (HCV) infection of Huh 7.5 cells. A Venn diagram depicting the number of genes that are differentially expressed (FDR > 13 and fold change ≥ 1.5) at 6, 48, and 72 hours after HCV JFH-1 infection are shown. Genes that were significantly differentially expressed at more than one time point are shown in the overlapping regions.

The 5' capped RNA samples from HCV infected and control cells were analyzed by RNA sequencing and data processed as described in Experimental Procedures. Bioinformatics analysis of the data identified 100, 684, and 1844 differentially expressed annotated genes in acutely infected, as compared to mock infected, Huh 7.5 cells at 6, 48, and 72 hours, respectively (fold change ≥ 1.50 and false discovery rate (FDR) > 13 (represents an untransformed FDR < 0.05 or 5 false positives out of 100) (Figure 1, Table 1 and Table 2) [14].

**Table 1:** The 50 most highly upregulated genes during acute HCV infection. The USeq DefinedRegionScanSeqs (DRSS) application was used to count the sequence reads for each annotated gene and score them for differential expression. The scores were controlled by multiple testing and ranked by transformed false discovery rate (FDR) and fold change (Experimental procedures). An FDR of 20 represents an untransformed FDR of 0.01 or 1 false positive per 100 observations and an FDR of 50 represents an untransformed FDR of 0.00001 or 1 false positive per 100,000 observations. Annotated genes that were significantly increased after acute HCV infection, relative to mock-infected cells, are presented in the order of their fold change. Newly identified differentially expressed genes are shown in bold. Genes marked with * had higher fold change at 48 h than at 72 h. A complete list of significantly upregulated genes is provided in Supplemental Table 1.

Ensembl Gene ID	Gene Name	Description	Fold Change	FDR
ENSG00000174951	FUT1	Fucosyltransferase 1	20.51	104.09
**ENSG00000130487**	**KLHDC7B**	**Kelch domain containing 7**	**18.93**	**141.94**
**ENSG00000136826**	**KLF4**	**Kruppel-like factor 4**	**15.10**	**142.63**
ENSG00000128591	FLNC	Filamin C	13.38	1160.20
ENSG00000148677	ANKRD1	Ankyrin repeat domain 1	12.06	351.26
ENSG00000205595	AREG	Amphiregulin	11.63	229.81
**ENSG00000135842**	**C1orf24**	**Chromosome 1 open reading frame 24 (C1orf24)**	**7.23**	**63.37**
**ENSG00000180535**	**MIST1**	**Xlass II bHLH protein MIST1**	**6.99**	**84.43**
**ENSG00000136383**	**ALPK3**	**Alpha-kinase 3**	**6.83**	**202.42**
**ENSG00000139269**	**INHBE**	**Inhibin, beta E**	**6.40**	**1324.33**
ENSG00000107731	UNC5B	Unc-5 homolog B (C. elegans)	6.17	265.31
ENSG00000049323	LTBP1	Latent transforming growth factor beta binding protein 1	6.14	129.49
**ENSG00000048052**	**HDAC9**	**Histone deacetylase 9**	**6.09**	**137.83**
**ENSG00000198205**	**ZXDA**	**Zinc finger, X-linked, duplicated A**	**6.02**	**47.22**
**ENSG00000111981**	**ULBP1**	**UL16 binding protein 1**	**6.01**	**37.66**
ENSG00000130513	GDF15	Growth differentiation factor 15	5.90	958.30
**ENSG00000122728**	**TAF1L**	**TAF1-like RNA polymerase II, TATA box binding protein (TBP)-associated factor**	**5.54**	**41.06**
**ENSG00000138670**	**RASGEF1B**	**RasGEF domain family, member 1B**	**5.41**	**39.54**
**ENSG00000176971**	**LOC387758**	**Similar to RIKEN cDNA 1110018M03**	**5.39**	**23.55**
**ENSG00000160999**	**APS**	**Adaptor protein with pleckstrin homology and src homology 2 domains**	**5.18**	**22.09**
ENSG00000006327	TNFRSF12A	Fucosyltransferase 1	5.13	286.27
**ENSG00000184545**	DUSP8*	**Dual specificity phosphatase 8**	**5.05**	**87.30**
ENSG00000130766	SESN2	Sestrin 2	4.84	593.42
ENSG00000070669	ASNS	Asparagine synthetase	4.80	577.30
ENSG00000136997	MYC	v-myc myelocytomatosis viral oncogene homolog	4.71	991.67
**ENSG00000168679**	**SLC16A4**	**Solute carrier family 16 (monocarboxylic acid transporters), member 4**	**4.65**	**24.23**
ENSG00000099889	ARVCF	Armadillo repeat gene deletes in velocardiofacial syndrome	4.53	240.65
**ENSG00000162772**	ATF3*	**Activating transcription factor 3**	**4.47**	**695.61**
**ENSG00000106948**	**AKNA**	**AT-hook transcription factor**	**4.45**	**773.28**
**ENSG00000167861**	**C17orf28**	**Chromosome 17 open reading frame 28**	**4.43**	**142.87**
**ENSG00000138685**	**FGF2**	**Fibroblast growth factor 2**	**4.08**	**23.78**
ENSG00000105327	BBC3	BCL2 binding component 3	4.06	230.60
ENSG00000111087	GLI1	Glioma-associated oncogene homolog 1 (zinc finger protein)	4.02	41.66
ENSG00000065911	MTHFD2	Methylenetetrahydrofolate dehydrogenase (NADP+ dependent) 2,	4.00	693.07
**ENSG00000115520**	**COQ10B**	**Coenzyme Q10 homolog B (S. cerevisiae)**	**3.96**	**22.43**
**ENSG00000105499**	PLA2G4C*	**Phospholipase A2, group IVC (cytosolic, calcium-independent)**	**3.92**	**44.14**
**ENSG00000065600**	**TMEM206**	**Transmembrane protein 206**	**3.90**	**57.11**
ENSG00000108551	RASD1	RAS, dexamethasone-induced 1	3.89	70.16
**ENSG00000152137**	**HSPB8**	**Heat shock 22kDa protein 8**	**3.87**	**64.97**
ENSG00000112182	BACH2	BTB and CNC homology 1, basic leucine zipper transcription factor 2	3.83	76.67
**ENSG00000157765**	**SLC34A2**	**Homo sapiens cDNA FLJ90534 fis, highly similar to Homo sapiens sodium dependent phosphate transporter isoform NaPi-3b mRNA**	**3.80**	**41.45**
**ENSG00000163393**	**SLC22A15**	**Solute carrier family 22 (organic cation transporter), member 15**	**3.77**	**104.50**
ENSG00000168003	SLC3A2	Solute carrier family 3 (activators of dibasic and neutral amino acid transport), member 2	3.77	1977.88
**ENSG00000164949**	**GEM**	**GTP binding protein overexpressed in skeletal muscle (GEM)**	**3.71**	**51.70**
**ENSG00000107281**	**NPDC1**	**Neural proliferation, differentiation and control, 1**	**3.68**	**38.82**
**ENSG00000154025**	**SLC5A10**	**Solute carrier family 5 (sodium/glucose cotransporter), member 10**	**3.67**	**50.35**
**ENSG00000196517**	**SLC6A9**	**Solute carrier family 6 (neurotransmitter transporter, glycine), member 9**	**3.65**	**153.70**
**ENSG00000166173**	**LARP6**	**La ribonuleoprotein domain family, member 6**	**3.62**	**146.96**
**ENSG00000184371**	**CSF1**	**Colony stimulating factor 1 (macrophage)**	**3.58**	**132.33**
ENSG00000120129	DUSP1	Dual specificity phosphatase 1 (DUSP1)	3.53	446.22

**Table 2:** The 25 most highly downregulated genes during acute HCV infection. Genes are presented in the order of fold change relative to controls and the time after HCV infection and FDR values are provided. Newly identified downregulated genes are shown in bold. A complete list of significantly upregulated genes are provided in Supplemental Materials.

Ensembl Gene ID	Gene Name	Description	Fold Change	FDR	Time Point
**ENSG00000196475**	**GK2**	**Glycerol kinase 2**	**−17.49**	**−22.37**	**72 h**
**ENSG00000164588**	**HCN1**	**Hyperpolarization activated cyclic nucleotide-gated potassium channel 1**	**−15.64**	**−23.39**	**6 h**
**ENSG00000101438**	**SLC32A1**	**Solute carrier family 32 (GABA vesicular transporter), member 1**	**−9.34**	**−68.18**	**72 h**
**ENSG00000178695**	**KCTD12**	**Potassium channel tetramerisation domain containing 12**	**−8.10**	**−41.92**	**6 h**
**ENSG00000182687**	**GALR2**	**Galanin receptor 2**	**−6.23**	**−17.14**	**6 h**
ENSG00000120738	EGR1	Early growth response 1	−5.33	−259.33	6 h
**ENSG00000170989**	**EDG1**	**Endothelial differentiation, sphingolipid G-protein-coupled receptor, 1**	**−4.20**	**−21.82**	**72 h**
**ENSG00000186074**	**NKIR**	**NK inhibitory receptor precursor**	**−4.10**	**−17.18**	**72 h**
**ENSG00000153266**	**ZNF312**	**Zinc finger protein 312**	**−3.91**	**−116.99**	**6 h**
**ENSG00000143595**	**AQP10**	**Aquaporin 10**	**−3.32**	**−97.38**	**72 h**
**ENSG00000153266**	**ZNF312**	**Zinc finger protein 312**	**−3.01**	**−71.64**	**72 h**
**ENSG00000167183**	**PRR15L**	**Proline rich 15-like**	**−2.95**	**−39.58**	**72 h**
ENSG00000131470	TBPIP	TBP-1 interacting protein (TBPIP)	−2.80	−17.88	6 h
**ENSG00000134389**	**CFHL5**	**Complement factor H-related 5**	**−2.72**	**0.37**	**48 h**
**ENSG00000156475**	**PPP2R2B**	**Protein phosphatase 2, regulatory subunit B (PR 52)**	**−2.65**	**−26.85**	**72 h**
**ENSG00000159261**	**CLDN14**	**Claudin 14 (CLDN14)**	**−2.58**	**−26.90**	**72 h**
**ENSG00000186910**	**SERPINA11**	**Serpin peptidase inhibitor, clade A (alpha-1 antiproteinase, antitrypsin)**	**−2.53**	**−25.05**	**72 h**
**ENSG00000182782**	**GPR109A**	**G protein-coupled receptor 109A**	**−2.50**	**−29.16**	**72 h**
**ENSG00000182782**	**GPR109B**	**G protein-coupled receptor 109B**	**−2.50**	**−29.16**	**72 h**
**ENSG00000197711**	**HP**	**Haptoglobin**	**−2.42**	**−291.19**	**72 h**
**ENSG00000197711**	**HPR**	**Haptoglobin-related protein**	**−2.42**	**−291.19**	**72 h**
**ENSG00000130427**	**EPO**	**Erythropoietin**	**−2.39**	**−44.50**	**72 h**
ENSG00000143224	PPOX	Protoporphyrinogen oxidase	−2.37	−15.81	6 h
ENSG00000162344	FGF19	Fibroblast growth factor 19	−2.31	−40.79	6 h

**Figure 2:**
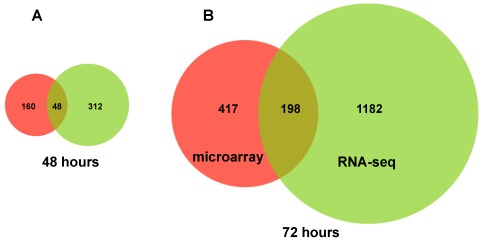
Significantly differentially expressed annotated genes as determined by RNA-seq analysis of 5' capped RNA compared to microarray analysis of poly(A)+ RNA (*p* > 0.05 and fold change of ≥2.0, [9]) after 48 (Panel A) and 72 hours of HCV infection of Huh 7.5 cells (Panel B).

The significantly differentially expressed genes in our RNA sequencing dataset were compared with a previously published comprehensive microarray dataset using a *p* value cutoff of <0.05 and fold change of 2 [9]. More than 80% of the differentially expressed genes at 48 and 72 hours in the RNA sequencing dataset were not significantly changed in the microarray analysis of poly(A) selected RNA of acutely HCV infected Huh 7.5 cells (Figure 2) [9]. Although minor differences in cell culture conditions might explain some of these differences, these results provide evidence that RNA sequencing analysis can provide new information regarding the changes in gene expression occurring during HCV infection. 

**Figure 3:**
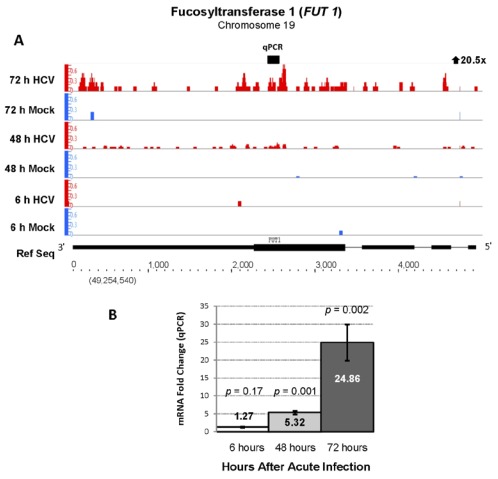
Increased expression of fucosyltransferase 1 (*FUT1*, Chr 19, − strand) during acute HCV infection. (**A**) RNA sequencing analysis of acutely HCV infected cells showed a 20.5-fold increase in ***FUT1*** gene expression with a highly significant FDR of 105 (represents <0.0000000001 false positives out of 100). Gene expression is displayed as the number of 36nt sequencing reads per kilobase of gene length per million reads (RPKMI) using the Integrated Genome Browser (IGB) (Experimental Procedures). The y-axis represents RPKMI, and x-axis represents chromosome location and gene structure. To present a better view of gene exon structure, the IGB sliced view that minimizes the size of intronic regions was used. RNA sequencing reads from a pool of three replicates of HCV infected cells at 6, 48 and 72 h are shown in red and mock-infected cells in blue. Gene structure (in kilobases), orientation and chromosomal location (gene start provided in parenthesis) are shown in the lower part of the panel next to Ref Seq. (**B**) Quantitative PCR (qPCR), although it measures only one specific region of transcript expression unlike sequencing analysis, was used to validate the RNA-seq results. Six mock infected and six HCV infected Huh 7.5 cell cultures (9.6 cm^2^) were analyzed by qPCR (n = 3 for each culture, total of n = 18) at each time point. qPCR analysis showed a 24.8-fold increase in mRNA at 72 hours after HCV infection. The qRT-PCR data represent the mean ± SEM and the location of qPCR primers is indicated in the IGB schematic in Panel A. *p*-values were calculated using the Student's t-test, and *p* values <0.05 were considered significant.

Approximately 37% of the genes that were significantly differentially expressed at 48 hours were also significantly changed at 72 hours. Highly differentially expressed transcripts that were newly identified or previously unvalidated were studied by qPCR analysis of six additional replicates of JFH‑1 infected Huh 7.5 cells (Figure 3–Figure 7, and Figures S1 to S4). 

The gene with the highest fold change (20-fold increase) in expression 48 and 72 hours after HCV infection was fucosyltransferase 1 (*FUT1*) which is involved in protein fucosylation and cell signaling (Figure 3, Panel A). A 24-fold increase in *FUT1* mRNA levels after 72 hours of infection was confirmed by qPCR analysis (Figure 3, Panel B). Previous microarray studies also showed upregulation of this transcript in HCV JFH1-J6 infected Huh 7.5 cells [9]. Significant increases in mRNA levels of glycosyltransferase genes involved in the synthesis of protein-bound and lipid-bound oligosaccharides were also identified, indicating that regulation of glycosylation pathways are affected by acute HCV infection (Supplemental Table 1). Since HCV envelope glycoproteins are modiﬁed by N-linked glycosylation, some of these gene products may be required for viral protein processing and regulation of their function.

**Figure 4:**
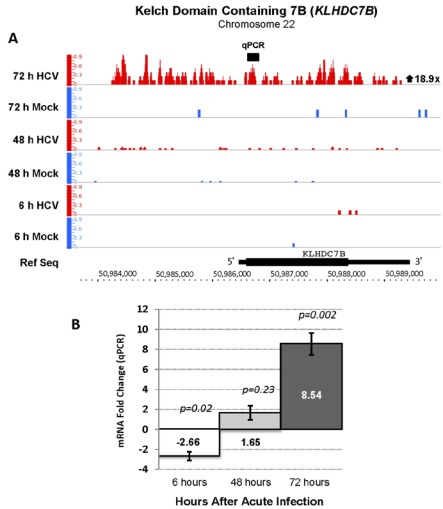
Increased expression of the Kelch domain containing 7B (*KLHDC7B*, Chr 22, + strand) gene during acute HCV infection. (**A**) The RNA-seq analysis identified an 18.9-fold increase *KLHDC7B* mRNA with an FDR value of 141.9. The gene structure, orientation and chromosomal location are shown below the sequencing reads. This example shows one benefit of analyzing 5' capped by RNA sequencing and provides evidence that an isoform of *KLHDC7B* mRNA with a longer 5' untranslated region (UTR) is expressed in Huh 7.5 cells after infection with HCV. There was no annotated gene or EST on the minus strand in this genomic region that provided an alternative explanation for the sequence reads observed upstream of the *KLHDC7B* 5' end. (**B**) qPCR analysis verified a major increase in expression of *KLHDC7B* and showed a 9-fold increase mRNA with the PCR primers used.

The Kelch domain containing 7B (*KLHDC7B*) gene, encodes a protein of unknown function that contains Kelch-repeat β-propellers that are known to undergo a variety of binding interactions with other proteins [21]. *KLHDC7B* was upregulated 19-fold in acutely HCV infected cells compared to controls (Figure 4, Panel A). Upregulation of this gene during acute HCV infection was not reported in previous studies [8,9,22]. The RNA-seq finding was confirmed by qPCR analysis with a fold change of 9 after 72 hours of infection (Figure 4, Panel B). 

**Figure 5:**
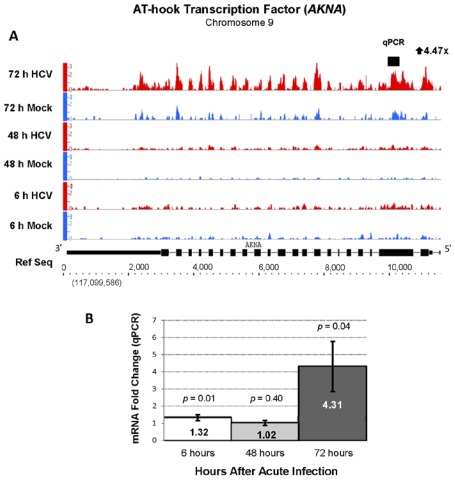
Increased expression of AT-hook transcription factor (*AKNA*, Chr 9, − strand) during acute HCV infection. (**A**) RNA sequencing analysis showed a 4.5-fold increase with an FDR of 773.3 and (**B**) qPCR showed a 4.3-fold increase in *AKNA* mRNA at 72hr after HCV infection.

Indicating a major reprogramming of host cell gene expression, a total of 276 genes involved in gene transcription were differentially expressed by 72 hours of acute HCV infection. Eighty percent of these genes were not previously determined differentially regulated during acute HCV infection. This included the upregulation of transcription factors regulating cell differentiation, inflammation and signal transduction including krupple-like factor 4 (*KLF4*, 15-fold), hexokinase domain containing 1 (*HKDC1*, 15-fold), ankyrin repeat domain 1 (*ANKRD1*, 12-fold), v-myc myelocytomatosis viral oncogene (*MYC*, 5-fold), and AT-hook transcription factor (*AKNA*, 4-fold) (Table 1). The upregulation of *AKNA*, *MYC*, and *ANKRD1 *mRNA transcripts was confirmed by qPCR (Figure 5 and Supplemental Figures 1 and 2, respectively). Two of these genes, not reported to be upregulated in previous studies, were *KLF4* and *AKNA*. The AT-hook transcription factors, of which* AKNA* is a member, repress or activate gene networks controlling inflammation, development, carcinogenesis, and metabolism are thought be critical in some disease states [23,24]. The finding of upregulation of *AKNA *expression raises the question that either the host or virus uses this change to downregulate a network of inflammatory genes during acute HCV infection.

**Figure 6:**
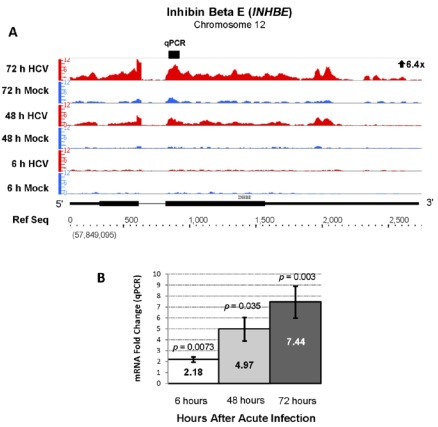
Increased expression of inhibin beta E (*INHBE*, Chr 12, + strand) during acute HCV infection. The RNA sequencing (6.4 fold increase, FDR = 1324.3) (**A**) and qPCR results (**B**) are presented as in Figure 3.

Our RNA sequencing analysis identified another biologically unpredicted pair of genes that were activated during acute HCV infection. Inhibin beta isoforms A and E (*INHBA* and *INHBE*) were upregulated 14 and 6-fold after acute HCV infection (Table 1, Figure 6). Upregulation of *INHBE* was confirmed increased by qPCR analysis with an average fold change of 7 (Figure 6, Panel B). Inhibin beta E (*INHBE*) is a member of the transforming growth factor (TGF-β) super family and plays a role in cell growth and proliferation [25]. *INHBE* is involved in regulation of human reproductive hormones and is dysregulated in endometrial and cervical carcinoma [25,26]. The upregulation of *INHBE* suggests that nonclassical TGF-β family member genes play regulatory roles during acute HCV infection. 

A number of studies have demonstrated the formation of membranous webs that include HCV replication complex proteins, during HCV infection [27,28]. Our RNA sequencing analysis revealed a 6-fold increase in *filamin C (FLNC)* mRNA, which produces a gene product that is critical in cross-linking actin filaments and attaching membrane glycoproteins proteins to the actin cytoskeleton (Supplemental Figure 3). Although increased *FLNC* was previously reported, we identified 128 additional members of the cytoskeleton gene family that were significantly differentially expressed during acute HCV infection. Ninety-six of these genes were not previously identified, including upregulation of *APS*, *SLC1A4*, *PSEN2*, *MAP1B*, and *PXK* (Supplemental Table 1). This data supports the previously reported role of actin and tubulin in formation of HCV replication complexes and RNA synthesis [27]. 

**Figure 7:**
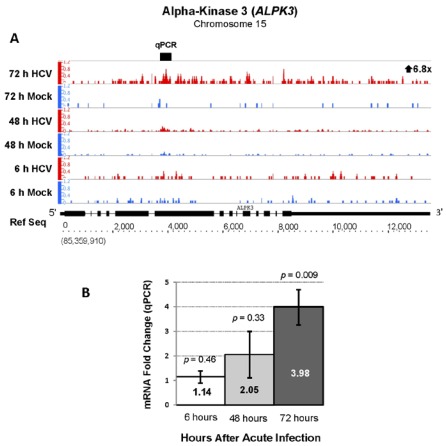
Increased expression of Alpha-kinase 3 (*ALPK3*, chr 15, + strand) during acute HCV infection. RNA sequencing analysis of acutely infected Huh 7.5 cells showed a 6.8 fold increase *ALPK3* mRNA with a highly significant FDR value of 202.4. (**A**) and qPCR analysis verified a significant increase (**B**).

RNA sequencing analysis also demonstrated a 6.8-fold upregulation (FDR of 202.4) of alpha-kinase 3 gene (*ALPK3*), a gene that may connect HCV RNA to the cytoskeleton, during acute HCV infection that was not previously reported. qPCR analysis confirmed upregulation of *ALPK3* with a 4-fold increase (Figure 7). *ALPK3* contains shares protein domains with *TRAF3IP1* (or MIP-T3) that binds microtubules, TRAF3 and associates with the HCV IRES in a riboproteomics study [29,30]. This suggests a possible mechanism for connecting HCV RNA to the cytoskeleton for regulatory purposes, such as replication complex formation [29,30]. 

### 2.3. Changes in Kegg Pathways during Acute HCV Infection

The RNA sequencing database of differentially expressed genes was analyzed to determine which of the 228 KEGG pathways were significantly enriched (altered) during acute HCV infection. MAPK signaling was the only KEGG pathway enriched at all times examined and had a progressive increase in the number of genes activated from 6 to 72 hours (Table 3). Other KEGG pathways enriched at both 48 and 72 hours post infection were adipocytokine signaling and TGFβ signaling. The TGFβ signaling pathway was previously reported to be deregulated during acute HCV infection and regulates cell cycle arrest, apoptosis, and fibrosis under experimental conditions [9]. The majority of enriched KEGG pathways were observed at 72 hours after infection. Although this included pathways reported in previous studies, such as apoptosis, sphingolipid metabolism and amino acid metabolism, there were a number of biologically relevant enriched pathways that we found in this analysis [8,9]. Novel KEGG pathways enriched at 72 hours included Insulin signaling pathway, RIG-I like receptor signaling, *H. pylori* pathway cytosoloic-DNA sensing pathway, extracellular matrix (EMC)-receptor pathway, NOD-like receptor signaling and Notch signaling. Significant alterations in RIG-I signaling gene expression were surprising since Huh 7.5 cells have a mutant RIG-I gene that impairs the interferon response. Some of the genes in this pathway may partly compensate for this RIG-I mutation. 

**Table 3:** Enriched Kegg pathways during acute HCV infection. Differentially expressed genes (DEGs) at each time point were analyzed with software that utilizes a Fisher’s exact test (*p* < 0.05 considered significant) to determine enriched pathways. The background gene list included only genes that were expressed (EGs), while genes that were not expressed were excluded from statistical analysis.

KEGG Pathway name	Genes in Pathway	6 h		48 h		72 h	
		DEGs (EGs)	*p* value	DEGs (EGs)	*p* value	DEGs (EGs)	*p* value
**MAPK signaling**	268	5 (168)	0.003	13 (173)	0.01	33 (177)	0.0005
**Cytokine-cytokine receptor interaction**	265			9 (90)	0.005	19 (92)	0.002
**Antigen processing and presentation**	78	2 (36)	0.02				
**RIG-I-like receptor signaling**	71					12 (44)	0.001
**Toll-like receptor signaling **	102					11 (58)	0.03
**NOD-like receptor signaling**	59					9 (40)	0.02
**Cytosolic DNA-sensing pathway**	56					7 (28)	0.02
**Epithelial cell signaling in *H. pylori* infection**	68					11 (53)	0.01
**Adipocytokine signaling**	68			7 (50)	0.002	13 (48)	0.0008
**TGF-beta signaling**	85			6 (68)	0.03	13 (55)	0.02
**Regulation of autophagy**	34					5 (18)	0.03
**Apoptosis**	89					12 (68)	0.04
**ECM-receptor interaction**	85			4 (37)	0.04		
**Jak-STAT signaling**	155					15 (77)	0.01
**Hepatitis C**	135					20 (98)	0.002
**mTOR signaling**	52	2 (41)	0.02				
**Hedgehog signaling**	56			5 (30)	0.004		
**Notch signaling**	47					9 (37)	0.01
**Renal cell carcinoma**	70	2 (58)	0.04				
**Basal cell carcinoma**	55			4 (31)	0.02		
**Hematopoietic cell lineage**	88			4 (29)	0.02		
**Osteoclast differentiation**	128			7 (76)	0.02	19 (76)	0.0001
**Insulin signaling**	138					19 (110)	0.01
**Type II diabetes mellitus**	48			4 (27)	0.01		
**Aminoacyl tRNA biosynthesis**	63					13 (41)	0.0001
**Ribosome biogenesis in eukaryotes**	81					12 (44)	0.001
**Sphingolipid metabolism**	40					9 (33)	0.005
**Glutathione metabolism**	50	2 (39)	0.03				
**Cyanoamino acid metabolism**	7	1 (4)	0.02				
**Ubiquinone and other terpenoid-quinone biosynthesis**	7	1 (5)	0.03				
**Taurine and hypotaurine metabolism**	10	1 (5)	0.03				
**Neurotophin signaling**	127					18 (94)	0.006
**One carbon pool by folate**	18					5 (17)	0.03
**Prion diseases**	36	2 (21)	0.006				

### 2.4. RNA Sequencing Analysis of 5' Capped RNA Identifies Upregulated Unannotated Pol II Transcripts

Using the open source software program Cufflinks we identified more than 1500 unannotated transcriptionally active regions in HCV infected Huh 7.5 cells that produced 5' capped RNA (data not shown) [31]. These regions were a minimum of 2 kilobases from well annotated genes in the Ensembl 62 (Hg19) database. Some of these transcripts may represent alternative 5' start sites or alternative 3' poly(A) sites of annotated genes. We further refined this list of unannotated transcripts to focus on RNAs that were differentially expressed in HCV infected compared to control cells after 48 and 72 hours of infection with an FDR of ≥13 and a fold change of 1.5. We also used an RPKM (reads per kilobase of gene exon length per million reads) cutoff of 5 to identify novel RNAs that were moderately to highly expressed (Table 4). 

**Table 4:** Unannotated transcripts increased during acute HCV infection. It displays ten unannotated transcripts with their respective chromosome location, transcript size, fold change and FDR. RPKM = reads per kilobase per million reads.

GENOME POSITION								
Chr	Start	Stop	Total BPs	HCV RPKM	MOCK RPKM	Fold Change	FDR	Time point
**8**	47528429	47529270	841	7.74	0.93	7.52	113.0	72 h
**5**	85865845	85866626	781	10.08	3.37	2.92	57.2	72 h
**4**	1384494	1385183	689	6.10	2.27	2.59	21.4	72 h
**8**	87354779	87355196	417	7.95	3.04	2.50	14.7	72 h
**16**	31580193	31580636	443	10.55	4.19	2.44	21.4	72 h
**17**	7967452	7968557	1105	6.15	2.62	2.33	101.1	48 h
**16**	21513065	21513758	693	8.71	3.94	2.17	21.9	72 h
**15**	64995200	64995463	263	31.29	15.07	2.07	95.1	48 h
**11**	122489643	122489913	270	9.48	4.76	1.97	18.1	48 h
**14**	65747331	65747885	554	11.64	6.17	1.86	15.4	72 h

**Figure 8:**
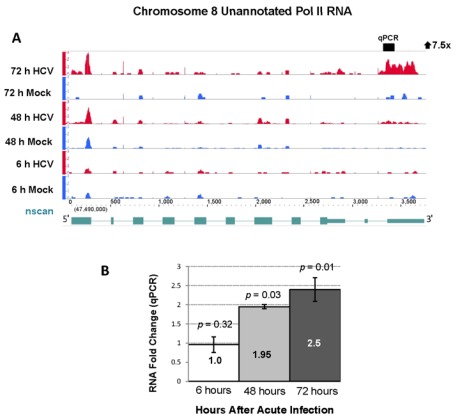
Differential expression of a novel Pol II transcript during acute HCV infection. (**A**) RNA sequencing analysis of 5' capped RNA showed a 7.5-fold increase in an unannotated RNA with a highly significant FDR of 113. The RNA transcript predicted by nscan is shown in lower part of Panel A in green [32]. (**B**) qPCR analysis showed a 2.5-fold increase in this unannotated Pol II RNA during acute HCV infection. The data are presented as in Figure 3.

One of the unannotated Pol II transcripts was upregulated by 5.0-fold at 48 and 7.5-fold at 72 hours after HCV infection and originated from a 39 kilobase region on chromosome 8 (Figure 8, panel A). qPCR analysis was performed on six additional replicates of HCV and mock infected cell cultures and showed a 2.5-fold increase in this RNA (*p*-value < 0.05) at the 72 hours (Figure 8, panel B).

Further bioinformatic analysis of this chromosome 8 region revealed that it has sequence homology to the recently identified asparagine synthetase pseudogene 1 (*ASNSP1*). We performed 5' and 3' rapid amplification of cDNA ends (RACE) to further characterize this Pol II RNA. The analysis showed that the Pol II RNA originating from chromosome 8 contains multiple splice variants with the largest variant extending beyond the boundaries of the recently identified pseudogene. Using the ORF Finder software (Softberry) we found a small peptide of 48 amino acids encoded in this unannotated transcript [33]. RACE sequence data confirmed that the novel RNAs were indeed originating from chromosome 8 and not from the parent gene on chromosome 7. We are not aware of other reports of pseudogenes exhibiting increased expression during acute HCV infection.

### 2.4. Effect of Gene Silencing of Selected Upregulated Genes on HCV JFH-1 Replication and Infectious Virus Production

The significant upregulation of at least 18 host genes involved in glycoprotein production and processing, such as *FUT1* (20–25-fold increased), was noted and raised the questions about the role of this pathway in the HCV life cycle (Supplemental Table 1). Fucosylation of viral proteins, such as influenza hemagglutinin, or cellular glycoproteins involved in Golgi-dependent processes as viral secretion, might be essential to HCV replication and the production of mature infectious virions [34,35]. Since the mRNA of the *FUT1* gene was so markedly upregulated, we determined if silencing of *FUT1* would alter HCV replication in the infectious cell culture system. In addition, since *KLHDC7B* was also highly upregulated during acute HCV infection (19-fold by RNA-seq) we also determined the effect of silencing this gene prior to HCV infection in Huh 7.5 cell cultures.

*FUT1* and *KLHDC7B *mRNA levels were downregulated by specific siRNAs as verified by qPCR analysis. *FUT1* mRNA was 46%, 35% and 26% of controls (cells transfected with scrambled siRNAs), and *KLHDC7B* mRNA level was 43%, 39% and 24% at 24, 48 and 72 hours after transfection, respectively (Figure 5S, Panel A). Control siRNAs did not downregulate FUT1 or KLHDC7B and had no significant cell toxicity (Figure 5S, Panel B).

**Figure 9:**
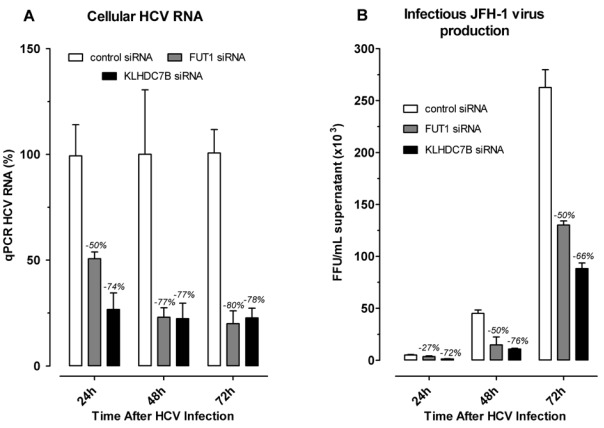
siRNA silencing of *FUT1* and *KLHDC7B *inhibit HCV replication and infectious virus production. Huh 7.5 cells were transfected with control siRNA (white bars), *FUT1* (gray bars) or *KLHDC7B* (black bars) and infected with JFH-1 (MOI = 0.5) for two hours, supplemented with fresh complete DMEM and continuously cultured to 24, 48, and 72 hours. At each time point, HCV RNA was measured by real-time PCR (**A**) and the titer of infectious HCV was measured by serial dilution of culture supernatants (**B**). The percent inhibition produced by each siRNA is shown above each bar in the graph.

24 hours after siRNA transfection, cells were infected with JFH-1 (MOI = 0.5). The effect of siRNA gene silencing on HCV replication was first measured by qPCR quantification of HCV RNA as described in Experimental procedures. HCV RNA levels were reduced by 50%, 77% and 80% in *FUT1 *and by 74%, 77% and 78% in *KLHDC7B* gene siRNA treated cells, relative to controls, at 24, 48 and 72 hours after HCV infection, respectively (Figure 9, Panel A). 

The effect of siRNA gene silencing of *FUT1 *and* KLHDC7B* on the production of infectious HCV particles in cell culture was also determined. Media collected from siRNA treated and HCV RNA transfected cells, plus controls, was assayed to determine the titer of infectious virus produced in each culture. The titer of infectious HCV produced in cell cultures with *FUT1 *silenced was decreased by 27%, 50% and 50% at 24, 48 and 72 hours after HCV infection, respectively (Figure 9, Panel B). Silencing of *KLHDC7B* decreased the titer of infectious HCV produced in cell culture by 72%, 76%, and 66% at the designated times relative to controls (Figure 9, Panel B). 

## 3. Discussion

We describe the first report of RNA sequencing analysis of 5' capped RNAs isolated from acutely HCV infected Huh 7.5 cells and provide evidence that this approach identifies many new annotated and unannotated genes that are differentially expressed during acute HCV infection. At 48 and 72 hours post infection more than 80% of significantly differentially expressed genes reported in our study were not identified in prior gene array analysis of acute HCV infection using the same fold change. We also present the first analysis of unannotated transcripts, some likely to be ncRNAs, that are differentially expressed during acute HCV infection and may play significant roles in the viral-host interactions. Follow up siRNA studies of two newly identified, highly upregulated genes, *FUT1* and *KLHDC7B*, provided evidence their expression during acute HCV infection is essential for the production of HCV RNA and infectious viral particles. Our results suggest that this general approach will have applications in studying viral-host interactions in both model systems and clinical biospecimens. Our studies were done in proliferative, standard cultures, of Huh 7.5 cells. It will be of interest to determine the similarities and differences in gene expression of acutely HCV infected proliferative and growth arrested differentiated human hepatoma-derived cells [36]. In comparison with our reported ENCODE tilling array (interrogates 1% of the human genome) analysis of HCV infected cirrhotic liver that identified 95 differentially expressed genes as compared to controls, our current analysis of acutely infected hepatoma cells showed that 20% of these genes were also differentially expressed [14]. This is a relatively large percentage of overlap since liver represents a much more complex transcriptome due to the presences of a number of other cell types besides hepatocytes and the fact that the ENCODE analysis was of chronically infected cirrhotic liver. Similar changes in genes involved in the immune response (e.g., *IRF1*, *RFX5* and *PSMB4*), cell structure (*FLNA*, *PLXNA3* and *CAPZA*) and growth (*CTGF* and *RHBDF1*) were observed in the prior and current studies, providing evidence that there is overlap in the host response to acute and chronic HCV infection and that the Huh 7.5 model can partly mimic *in vivo *processes occurring during HCV infection. The results also indicate that transcriptome changes in acutely infected Huh 7.5 cells become more profound with propagation of infection. This suggests that as a greater percentage of cells becomes infected that the biological effects become more pronounced, raising interesting questions regarding the pathobiology in selected patients with hepatitis C.

### 3.1. Acute HCV Infection Reprograms Gene Transcription of Infected Cells

Many genes regulating transcription, representing approximately 20% of all genes differentially expressed at 72 h, showed major changes in expression during acute HCV infection. Although changes in interferon stimulated and other genes during HCV infection, activated initially via PAMP (pathogen associated molecular patterns) pathways such as RIG-I and Toll-like receptors, have been well described during HCV infection this study identifies additional gene networks that are stimulated or inhibited during acute infection. This included a dramatic 10–20 fold increase in *KLF4* and *ANKRD1* mRNA and a 4-fold increase in *AKNA*, mRNA indicating that the gene networks they regulate undergo major changes during acute HCV infection. 

A number of members of the Sp1-like/KLF family of transcription factors (*SP1*, *SP5*, *KLF10*, *KLF11*, *KLF13*, *KLF15*, *KLF16*, *KLF3*, *KLF6*, *KLF9*) were significantly upregulated during acute HCV infection. They regulate the biological processes of cell growth and morphogenesis [37]. *KLF4* was previously shown to play a crucial role in early development by regulating cell differentiation, tumor suppression, and steam cells [37,38]. Although changes in *KLF4* expression has not been reported during acute HCV infection, it has been found to be upregulated in macrophages infected with Sendai virus, bronchial epithelial cells infected with RSV, and in endothelial cells exposed to Candida albicans blastospores [39,40,41]. 

*AKNA* is predominantly expressed by lymphoid tissues and encodes a protein that binds to DNA by a conserved nine amino acid domain called AT-hook, which has been implicated in regulating gene transcription by altering chromatin structure. Studies have provided evidence that *AKN*A plays a critical role in regulating immune responses by activating the expression of the CD40 receptor and its ligand CD40L/CD154, two lymphocyte cell surface molecules critical for antigen-dependent-B-cell development [42]. Recent studies showed that *AKNA* is a major modulator of the intensity of *in vivo* inflammatory responses [23]. Knocking out *AKNA* sensitizes mice to pathogen-induced inflammation and caused sudden neonatal death due to systemic inflammation, especially in the lung, induced by inflammatory cytokines (interferon-1β, interferon-1γ) and proteins (neutrophilic granule protein, cathelin-related antimicrobial peptide) [23]. *AKNA *has not been previously reported to be differentially regulated during viral infections. Our observation suggests that the *AKNA* regulated gene network suppresses the innate response during acute HCV infection and deserves follow up studies. 

In addition to genes regulating transcriptional and translational processes, genes involved in RNA catabolism, both promoting (such as *UPF1*, *UPF3B* and *SMG1*) and inhibiting RNA degradation (*RNH*, *GSTP1* and *TNKS1BP*) were significantly upregulated during acute HCV infection. To establish and maintain infection, RNA viruses must escape the host cell RNA decay machinery [43]. Some of the genes showing significant increases in mRNA during acute HCV infection (such as *HuR*, *PCBP2*) encode proteins that were reported as critical in protecting Sindbis and Polio viruses from host cell driven RNA decay [44,45]. Genes involved in stabilizing cellular mRNAs, such as *PCBP4* (poly(rC) binding protein 4) and *PCBP1*, were also significantly upregulated. Previous reports have shown *PCBP2* is hijacked by viruses (such as polio, coxsackie and hepatitis A virus) and used to promote optimal viral translation and gene expression [46,47,48,49]. In addition, *PCBP2* and *PCBP* isoforms are also found in stress granules and play a role in 3' end mRNA processing [48,49]. Other differentially expressed genes that are part of RNA decay pathways that were not previously known to be altered during HCV infection were identified. They include *HNRPU* (2.2-fold increase; participate in VSV-mediated shutoff of host RNA metabolism [47]), *RNH1* (2-fold increase; RNA decay regulation), *UPF3B* (2-fold increase; nonsense mediated mRNA decay), *DCP2* (1.6-fold increase; mRNA-decapping), *XRN2* (1.6-fold increase; 5'-3' exonuclease), *RNASEH1* (1.6-fold increase; mRNA decay), *PABPC1* and *PABPC3* (both 1.5-fold increase; involved in mRNA deadenylation) [43,50]. 

*ALPK3*, upregulated by 4 to 7-fold our analysis and not previously reported, is a member of a unique family of atypical protein kinases implicated in regulating a number of cellular processes including mRNA translation [51]. Upregulation of *ALPK3 *has been described in acute infection of dendritic cells during with measles virus [52]. The protein encoded by *ALPK3* contains at least three protein domains [53]: an alpha-kinase domain at the C-terminus, imunoglobulin domain and immunoglobulin I-set domain. It also shares a microtubule interacting protein domain that associates with TNF receptor-associated factor 3 (TRAF3), and is found in *TRAF3IP1 *(or* MIPT3*) [29]. TRAF3 is an ubiquitin ligase and plays a critical role in activation of the innate immune response by regulating signal transduction from pathogen recognition receptors [23]. A recent study showed that MIP-T3 is a negative regulator of the type I IFN response by activators including RIG-I, MDA5 and TLR3 [54]. Moreover, MIP-T3 expression suppresses the activation of IFN-stimulated response elements in a dose-dependent manner [54]. Since HCV is known to impair the host innate immune response, *ALPK3* upregulation may represent an additional viral mechanism for impairing host innate immunity and facilitating chronic HCV infection. The increase in *ALPK3* may also facilitate 5' cap-independent HCV RNA translation or replication. A protein domain shared by alpha protein kinase-3 (ALPK-3) and MIP-T3 was found in a RNP complex with the HCV IRES [30]. One possibility is that ALPK-3 could join the HCV RNA in replication complexes to the cytoskeleton and membranous webs [28]. 

### 3.2. Acute HCV Infection Related Kegg Pathways

RNA sequencing analysis identified several host response pathways, including adipocytokine signaling, insulin signaling, and RIG-I signaling, that were not identified in prior analysis of acutely HCV infected Huh 7.5 cells [9,10]. TGF-beta signaling was activated, as in gene array studies of acute HCV infection, which has been implicated in pathological effects of chronic infection [9,22]. 

More than ten genes associated with RIG-I signaling were increased during acute HCV infection in our analysis. This was surprising in view of the mutation in RIG-I in Huh 7.5 cells that ablates RIG-I signaling [55]. The upregulation of multiple genes downstream from *IPS-1* (*G1P2*, 3.4-fold; *IL8*, 2.9-fold; *TRAF3*, 1.9-fold, and *TRAF2*, 1.9-fold) suggests crosstalk between other molecular pathogen recognition receptors and the RIG-I pathway in Huh 7.5 cells. 

Previous reports provide evidence that HCV perturbs glucose metabolism, leading to insulin resistance that accelerates liver fibrogenesis, impairs the response to interferon treatment and increases the risk of HCC [56]. Our analysis found additional genes upregulated during acute HCV infection, not previously identified, such as *HKDC1* (14.7-fold), *IRS2* (2.9-fold), *RHOQ* (2.2-fold), *SKIP*, (1.9-fold), *IRS1* (1.6-fold) and insulin receptor substrate-2 (*IRS2*), the main effector of insulin signaling in the liver [57] (Supplemental Figure 4). Since *IRS-2* is involved in activation of lipogenic genes, it may play a role in the formation of lipid droplets on which HCV replication complexes assemble [58,59]. *IRS2* overexpression has also been reported in the early phase of murine liver tumorigenesis and in human HCC pathological specimens and HCC lines [59]. 

Approximately 200 genes were found to be differentially expressed by RNA-seq analysis are involved in embryonic development and more than seventy percent of them were not previously reported to be differentially expressed during acute HCV infection. They include *FGF2* and *FZD9* (both 4-fold increase), *CEBPB* (2.7-fold increase), *LNK*, *MAFG* and *TFEB* (all 2.5-fold increase), *PKD1* and *CELSR1* (both 2.3-fold increase), *JAG2* and *NOTCH1* (both 2.1-fold increase). We provide the first evidence that the Hedgehog and Notch signaling pathways are increased during acute HCV infection. The Hedgehog pathway is a critical regulator of embryonic development, is increased in chronic HCV and HBV hepatitis, and plays a key role in fibrogenesis and the development of HCC [60,61,62]. A recent study provides evidence that Hedgehog signaling also plays a role in creating an environment supportive of HCV replication in cell culture [63]. These results provide evidence for the Hedgehog signaling pathway playing a role in during the establishment of HCV infection and its long term pathological effects during chronic infection.

### 3.3. HCV and Cellular Glycosylation Pathways

A number of host genes involved in protein glycosylation were significantly increased and not previously reported to be changed during acute HCV infection. In particular, a dramatic 21 to 25-fold increase in fucosyltransferase 1 mRNA was observed at 72 hours after infection. Although, an increase in *FUT1* expression has been reported in previous studies its effect on HCV replication remained unknown. *FUT1* has been noted to be upregulated, most likely by a viral induced transcriptional change, during acute CMV infection and was suggested to play a role in virus spreading among cells or possibly viral escape from immune responses [64]. In this study we show that silencing *FUT1* in Huh 7.5 cells inhibited HCV RNA replication by up to 80% of controls and infectious virion production was inhibited by 50%. These results provide direct evidence that the dramatic induction of *FUT1* does have a biological role during acute HCV infection and encourage follow up studies regarding the mechanism of this *FUT1* induction and its function during acute infection. 

In addition to a role in early viral replication, changes in protein glycosylation also play an important role in the pathogenesis and progression of some liver diseases [65,66]. Altered glycosylation is recognized as a core characteristic of many cancer cells and changes in glycan structures have been used in the development of biomarkers for liver damage [65,66]. Fucosylation is one of the most important glycosylation events in cancer cells; this pathway includes eleven known fucosyltransferases, as well as GDP-fucose synthetic enzymes and a GDP-fucose transporter [67]. Our study revealed increased expression of the *FUT1 *(21 to 25-fold) (α1,2 fucosyltransferases), *FUT5* (5-fold) (α1,3/α1,4), and *FUT8* (2-fold) (α1,6 or core fucose). In addition, genes involved in *de novo* GDP-fucose synthesis pathway as well as a salvage pathway showed increased expression (*FUK* and *TSTA3*) at 48 hours. Well-known fucosyltransferase products are sialyl selectin receptors (Lewis glycol-epitopes) that play a critical role in leukocyte extravasation, lymphocyte maturation, and cell signaling and development [67]. HIV, CMV, VZV and HSV-1 infections induce expression of Lewis X and Lewis Y epitopes by activating transcription of FUT genes, which play a vital role in the spread of these viruses [64,68]. A similar strategy of hijacking the selectin-dependent mechanism (binding of sLeX on tumor cells to P-selectin on endothelial cells) was reported during metastasis of several tumors [69]. Recent studies provide evidence that HSV-1 induction of specific FUTs (*FUT3*, *FUT5*, and *FUT6*) was initiated by interactions between viral dsRNA and PKR and RIG-I. It was also reported that the herpesviruses induced increases in Lewis antigens and the sialylation of other glycoproteins (CD45) regulate TCR signaling, adhesion and apoptosis in thymocytes [70,71,72]. 

### 3.4. KLDC7B Gene Silencing Inhibits HCV Replication

Regarding* KLHDC7B*, not normally expressed in human liver cells, one of our new findings was that it was highly upregulated (9 to 19-fold) during acute HCV infection. Moreover, siRNA silencing of *KLHDC7B* in cell cultures inhibited HCV RNA replication and the production of infectious virus. *KLHDC7B* contains six kelch motifs that are found in a number of proteins, some with roles during other infectious conditions [21]. Proteins with kelch-repeat domains bind other proteins, notably actin filaments [21].

Mining the GEO database [73] we found that *KLHDC7B* mRNA was increased under a number of biological conditions with most of them due to infectious agents. *KLHDC7B* was upregulated during vulvar intraepithelial neoplasia due to human papillomaviruses [74] and in dendritic cells infected with Chlamydia pneumonia [75]. A role for *KLHDC7B* during acute infections is further suggested by its upregulation in dendritic cells infected with Aspergillus fumigatus [76] and bronchial epithelial cells during acute RSV infection [77]. *KLHDC7B* expression is also induced by interferon gamma, TNF-α and IL-4 [78]. Although the function of *KLHDC7B* remains unknown, our results provide the first evidence that changes in *KLHDC7B* expression have significant effects on acute HCV infection. In view of the proposed role of *KLHDC7B* in actin binding [79], we speculate that it has a role in cytoskeleton and membrane rearrangements required for efficient HCV RNA replication and possibly secretion of infectious virus. The specific function of increases in *KLHDC7B* during acute HCV infection will require further investigation. 

## 4. Experimental Procedures

### 4.1. Cell Culture

The human hepatoma cell line, Huh 7.5, was generously provided by Charles M. Rice and was maintained in Dulbecco’s modified Eagle’s medium (DMEM) (Invitrogen) supplemented with 100 U/mL of penicillin, 100 µg/mL of streptomycin, nonessential amino acids, and 10% fetal bovine serum (FBS) (Invitrogen) at 37 °C in 5% CO_2_ [80]. 

### 4.2. Production of Infectious HCV

Hepatitis C virus was generated from construct HCV pJFH1 that was kindly provided by Wakita [81]. *In vitro* transcribed HCV JFH1 RNA was electroporated into Huh 7.5 cells and the transfected Huh 7.5 cells were maintained and sub-cultured every three days. The titer of infectious HCV was determined by immunofluorescence assays with anti-NS5A antibody [82]. The viral titer is expressed as focus-forming units per milliliter (ffu/mL). Once the viral titer reached to 8.0 × 10^4^ ffu/mL, the medium containing Hepatitis C virus was stocked at −80 °C as a stock. 

### 4.3. Infection of Cells with HCV JFH1

Huh 7.5 cells were seeded at 2.5 × 10^5^/well in 6-well plates (9.6 cm^2^ per well). After 18 hours of culture, the cells were infected with HCV at an MOI of 0.5. The inoculums were incubated with cells for two hours at 37 °C and then supplemented with fresh complete DMEM. The cells were then continuously cultured to 6, 48, and 72 hours. Controls were Huh 7.5 cells grown under identical conditions and mock infected (conditioned media) for the same time. These time points were selected based on previous microarray studies showing maximal changes in differential gene expression after 48 and 72 hours of acute JFH1 HCV infection [8,9]. The six hour time point was chosen as an interval when few gene expression differences would be observed between HCV and mock-infected cells. 

### 4.4. RNA Isolation

Total RNA was purified from infected and mock-infected Huh 7.5 cells using Trizol (Invitrogen, Grand Island, NY, USA) as described previously [14]. RNA quality was assessed using a Bioanalyzer (Agilent, Santa Clara, CA, USA) and all samples had a minimum RIN of 9.0. Equal amounts of total RNA were pooled from three cultures (9.6 cm^2^) for each condition and time point and 5' capped RNA was purified using a recombinant GST fusion high-affinity variant of eIF4E (eIF4E_K119A_) which binds m^7^Gpp with a ten‑fold higher affinity compared to wild-type eIF4E [12,17,83]. Six additional cultures (9.6 cm^2^) for each condition and time point were used to isolate total RNA for qPCR validation studies. The quantity of all RNA samples was measured by NanoDrop analysis and its integrity confirmed with a Bioanalyzer.

### 4.5. RNA Sequencing

cDNA libraries were prepared from the 5' capped RNA samples using random hexamers following the Illumina RNA sequencing protocol [16]. Single 36bp RNA-Seq reads were obtained as previously described (Illumina GA II) [16]. A total of 17.2–17.6, 32.8–36.6 and 10.2–16.9 million sequencing reads were obtained for the 6, 48 and 72 hour HCV infected and control samples, respectively. Bioinformatic analysis of the sequencing data included adjustments for the depth of sequencing at different times (see below). 

### 4.6. Bioinformatics

RNA sequencing reads were aligned to the February 2009 human reference sequence genome (GRCh37/hg19) using the Bowtie short read aligner [84]. Visualization tracks were prepared for each of the samples using the USeq ReadCoverage application; these tracks can be viewed using the Integrated Genome Browser (IGB) [85]. 

Analysis to identify differential expression of genes began with the Ensembl list of known genes. The USeq DefinedRegionScanSeqs (DRSS) application was used to count the reads intersecting exons of each annotated gene and score them for differential expression in both infected and mock-infected cells [86]. These scores are controlled for multiple testing and ranked by false discovery rate (FDR) and normalized ratio. The criteria for designating individual genes as significantly differentially expressed was set at a transformed false discovery rate (designated FDR) of >13 (represents an untransformed FDR <0.05 or <5 false positives per 100 observations) and normalized change of ≥1.5 fold relative to controls [14]. Note: an FDR of 20 represents an untransformed FDR of 0.01 or 1 false positive per 100 observations and an FDR of 50 represents an untransformed FDR of 0.00001 or 1 false positive per 100,000 observations.

All gene ratios are based on reads per kilobase per million reads (RPKM), to correct for gene length and total reads per sample, as previously reported [87]. We also developed a novel approach to further refine this normalization strategy by employing reads per kilobase per million reads interrogated (RPKMI) which replaces the total number of aligned reads with the number of reads matching a list of known genes. This novel method corrects for disproportionate intronic and non-genic reads between RNA samples, which may be a significant confounder using the RPKM method. This method was used to develop read tracks that can be visualized in the Integrated Genome Browser (IGB) [85].

We designed custom software to identify enriched KEGG pathways using a Fisher’s exact test and display differentially expressed genes at each time-point. 

Unannotated transcripts were identified using the Cufflinks application [31]. Differential expression of identified novel regions was determined using the DRSS application as described above. We used an RPKM cutoff of 5 for follow-up by qPCR and RACE analysis.

### 4.7. Real-Time PCR (qPCR)

Six mock infected and six HCV infected Huh 7.5 culture specimens were used for further analysis of differentially expressed genes at each time point. Total RNA from infected and control hepatocytes were extracted using TRIzol. First-strand cDNA was synthesized using Moloney Murine Leukemia Virus reverse transcriptase (SuperScript III; Invitrogen) with 20 ng/mL of RNA at 55 °C (60 min) with random hexamer primers. Each qPCR reaction was carried out in a 384-well optical plate (Roche Applied Science) in a 10 µL reaction buffer containing LightCycler 480 Probes Master Mix (100 mM Tris-HCl, 100 mM KCl, 400 mM of each dNTP (with dUTP instead of dTTP), 64 mM MgCl_2_, FastStart Taq DNA Polymerase, 0.3 mM of each primer, 0.1 mM hydrolysis probe and approximately 50 ng of cDNA (done in triplicate)). Triplicate incubations without template were used as negative controls. Thermal cycling was done in a Roche LightCycler 480 System (Roche Applied Science). The qPCR thermo cycling was 95 °C for 5 min, 45 cycles at 95 °C for 10 s, 59 °C for 30 s and 72 °C for 1 second. The relative quantity of each RNA transcript was calculated with the comparative Ct (cycling threshold) method using the formula 2ΔCt. ΔCt represents the difference between target gene expression in mock-infected samples and target gene expression in HCV-infected samples. Reference genes (GAPDH and β-actin, ACTB) were used as controls and statistical significance was evaluated using the Student’s t-test.

4.8. 5' and 3' RACE Analysis 

To define novel RNA transcript structure from the differentially expressed unannotated regions of chromosomes 8, 5' and 3' rapid amplification of cDNA ends (RACE). We designed gene-specific primers on the minus and plus strand of five discrete regions (putative exons) on chromosome 8 that were found to upregulated in acute HCV infection by RNA-Seq analysis. To verify the chromosome location of each RACE PCR product, each PCR product was gel purified and cloned using a TA cloning vector. Cloned products were then sequenced with an Applied Biosystems 3130xl Genetic Analyzer.

### 4.9. RNA Interference

For transient transfection, chemically synthesized 21-nucleotide sense and antisense RNA oligonucleotides against *FUT1* and *KLHDC7B* and control nontargeting (scrambled) RNA were purchased from Santa Cruz Biotechnology. Huh 7.5 cells were plated on six-well plates at 150,000 cells per well and the next day transfected with siRNA at a final concentration of 33 nM per well using Lipofectamine^TM^ 2000 (Invitrogen), according to manufacturer’s protocols. Efficiency of gene silencing was verified by real-time PCR. To study the effect of selected gene knock-down on HCV production, siRNA transfected cells were infected with JFH-1 at the *MOI* of 0.5 for two hours at 37 °C, then washed with PBS and supplemented with fresh complete DMEM. The cells were then continuously cultured to 24, 48, and 72 hours. At each time point, cell-free supernatants and cells were harvested for assessment of HCV production (HCV RNA and infectious particles, see below). Experiment was done in triplicate and performed two times. Cytotoxicity assays were done under conditions that mimicked the siRNA studies. Huh 7.5 cells were plated in 96-well plate, cultured for 24 hours and transfected with siRNA against FUT1, KLHDC7B or control siRNA. Cells were mock infected 24 hours after transfection. Cell supernatants were collected (72 hours after transfection) and assayed for cellular toxicity using a lactate dehydrogenase cytotoxicity assay kit, CytoTox-ONE (Promega, Madison, WI, USA) according to manufacturer’s recommendations.

### 4.10. Quantification of Infectious HCV Virions and HCV RNA

Viral titers were determined by limiting dilution analysis of culture supernatants, as described before [82,88]. Briefly, medium from each sample in the siRNA transfection/JFH-1 infection experiments was collected and stored at −20 °C. Cell supernatants were serially diluted 10-fold in complete DMEM. Naive Huh 7.5 cells were grown on cover slips in 24-well plates and infected with the transfection sample medium for 2h at 37 °C and then supplemented with fresh complete DMEM. The level of HCV infection was determined 48 hours post-infection by immunofluorescence staining for HCV NS5A. 

For immunofluorescence assay, cells were extensively washed with PBS, fixed with 4% paraformaldehyde, and permeabilized with 0.2% Triton X-100. Fixed cells were blocked with 1% bovine serum albumin and 1% normal goat serum in PBS. HCV NS5A protein was detected in cells by incubation with an NS5A-specific monoclonal antibody and visualized with the secondary goat anti‑mouse IgG conjugated with Alexa Fluor 594 fluorescein (Invitrogen, 1:1000 dilutions). Cover slips were mounted onto slides with DAPI (Vector labs), and the HCV NS5A were visualized by fluorescence microscopy (Nikon E400). The viral titer is expressed as focus-forming units per milliliter of supernatant (ffu/mL), as determined by the average number of NS5A-positive foci detected by immunofluorescence for NS5A.

For HCV RNA quantification, total cellular RNA was harvested and purified using Trizol Reagent (Invitrogen), reverse transcribed to cDNA using SuperScript III; Invitrogen. Real-time PCR was performed using Universal Probe Library (as described above) and the JFH-1 5'UTR specific primers 5'-CATGGCGTTAGTATGAGTGTCg-3' and 5'-GGTTCCGCAGACCACTATG-3'.

## 5. Conclusions

RNA sequencing analysis of differentially selected RNAs demonstrated that acute HCV infection causes wide-ranging effects on the expression of annotated and unannotated Pol II genes. This approach is likely to have general applications in understanding the host response and viral induced pathology by more comprehensively examining selected biospecimens. We provide direct evidence, using siRNA gene silencing, that FUT1 and KLHDC7B are required for HCV replication and production of infectious virus during acute HCV infection. 

## Supplementary Files

Supplementary File 1: PDF-Document (PDF, 1210 KB)

Supplementary File 2: XLS-Document (XLS, 484 KB)
