# Supplementary material for: RNA-Sequencing Analysis of 5' Capped RNAs Identifies Many New Differentially Expressed Genes in Acute Hepatitis C Virus Infection
Source: Viruses. 2012 Apr 16;4(4):581–612. doi: 10.3390/v4040581 (PMC3347324; doi:10.3390/v4040581)

Figure S1. Differential expression of c-myc in HCV infected Huh7.5 cells as compared to mock-infected cells. (**A**) Gene expression is displayed by number of reads per kilobase of gene length per million reads (RPKM) using the Integrated Genome Browser (IGB). The y-axis represents RPKM, and x-axis represents chromosome location and gene structure (Methods). RNA sequencing reads from a pool of three replicates in HCV infected cells at 6, 48 and 72h are shown in red, and in mock-infected cells in blue. Gene structure, orientation and chromosomal location are shown in black. (**B**) Quantitative PCR was used to investigate (and check the RNA-seq data) the effect of JFH-1 infection on Huh7.5 cell gene expression. Six mock infected and six HCV infected hepatocyte culture specimens were used for qPCR at each time point. The qRT-PCR data shown represent the means ± standard error (SEM) for triplicate samples and the location of qPCR primers is indicated on the IGB plot (left side). *p*-values were calculated using the Student's t-test, and *p* values <0.05 were considered significant.

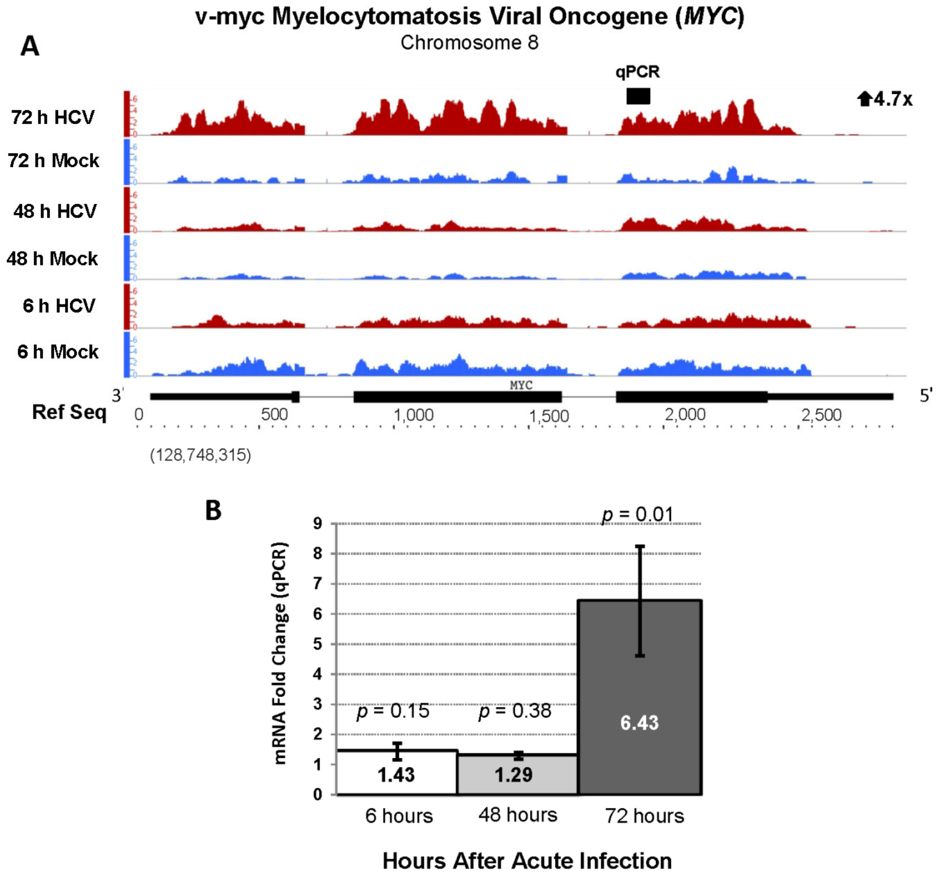

Figure S2. Differential expression of ANKRD1 in HCV infected Huh7.5 cells as compared to mock-infected cells. Our RNA-seq data identified a fold change of 12.1 and FDR value of 351.2 (Panel **A**, sequencing reads displayed in IGB) and qPCR assays indicated a fold change of 4.7 (Panel **B**, qPCR results). The RNA sequencing and qPCR data are presented as in Supplemental Figure 1.

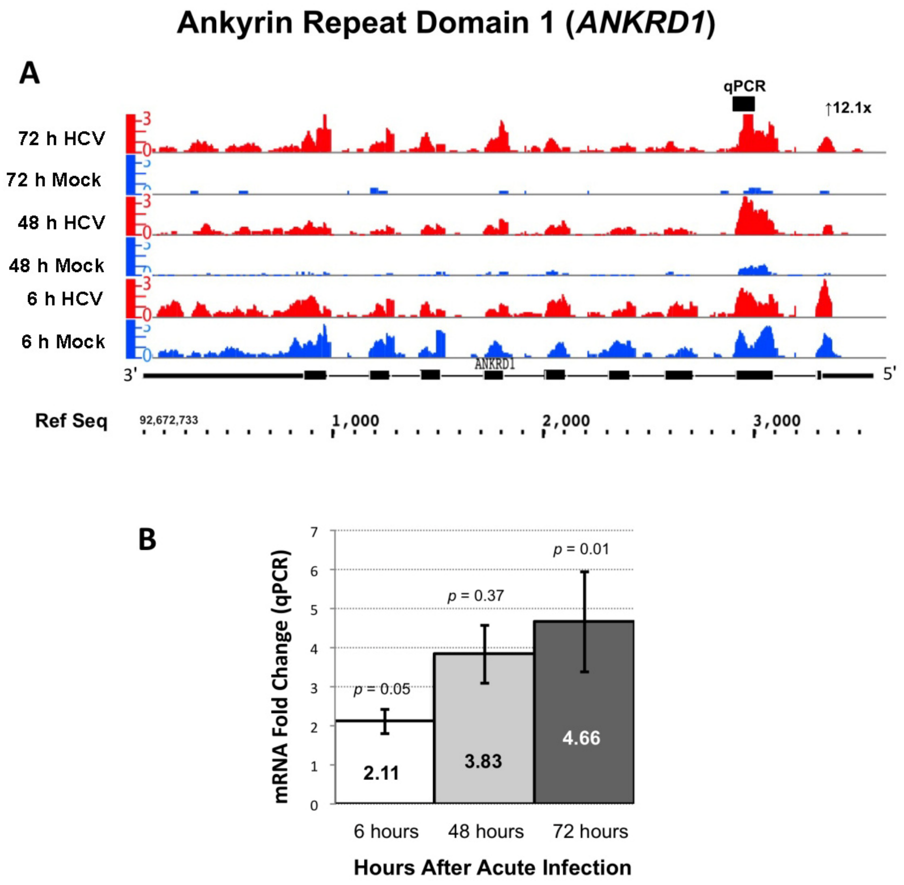

Figure S3. Differential expression of Filamin C in HCV infected Huh7.5 cells as compared to mock-infected cells. Our RNA-seq data identified a fold change of 13.4 and FDR value of 1160.2 (Panel **A**, sequencing reads displayed in IGB) and qPCR assays indicated a fold change of 5.2 (Panel **B**, qPCR results). The RNA sequencing and qPCR data are presented as in Supplemental Figure 1.

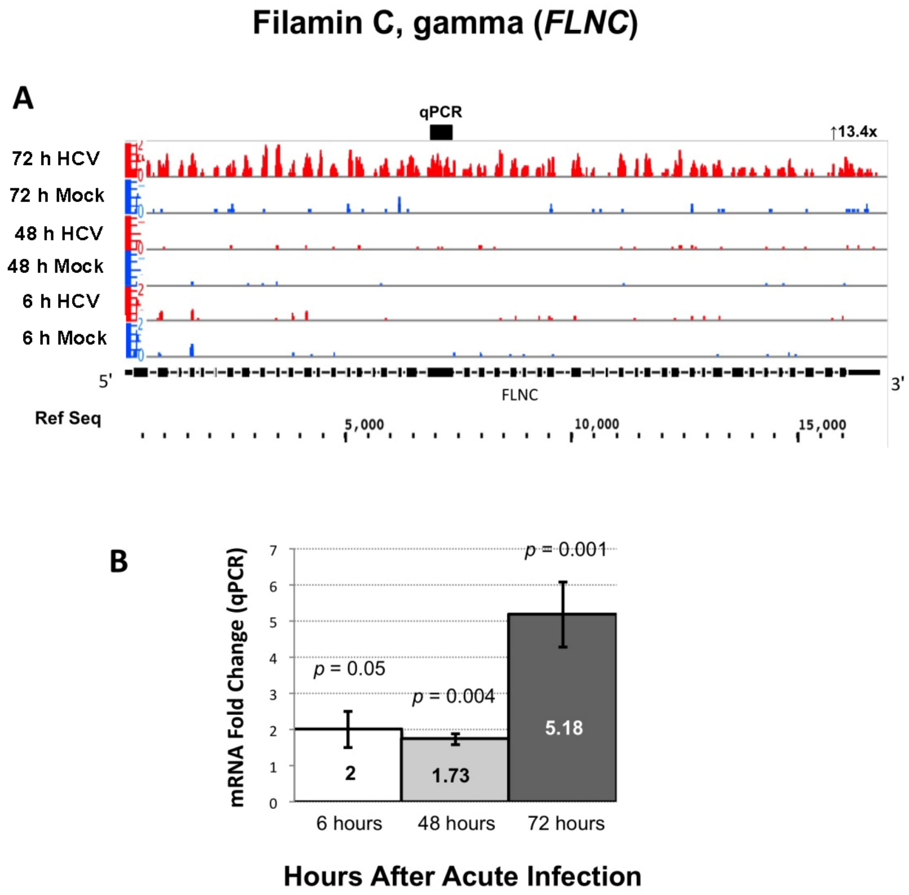

Figure S4. Differential expression of IRS-2 in HCV infected Huh7.5 cells as compared to mock-infected cells. Our RNA-seq data identified a fold change of 2.86 and FDR value of 133.9 (Panel **A**, sequencing reads displayed in IGB) and qPCR assays indicated a fold change of 3.8 (Panel **B**, qPCR results). The RNA sequencing and qPCR data are presented as in Supplemental Figure 1.

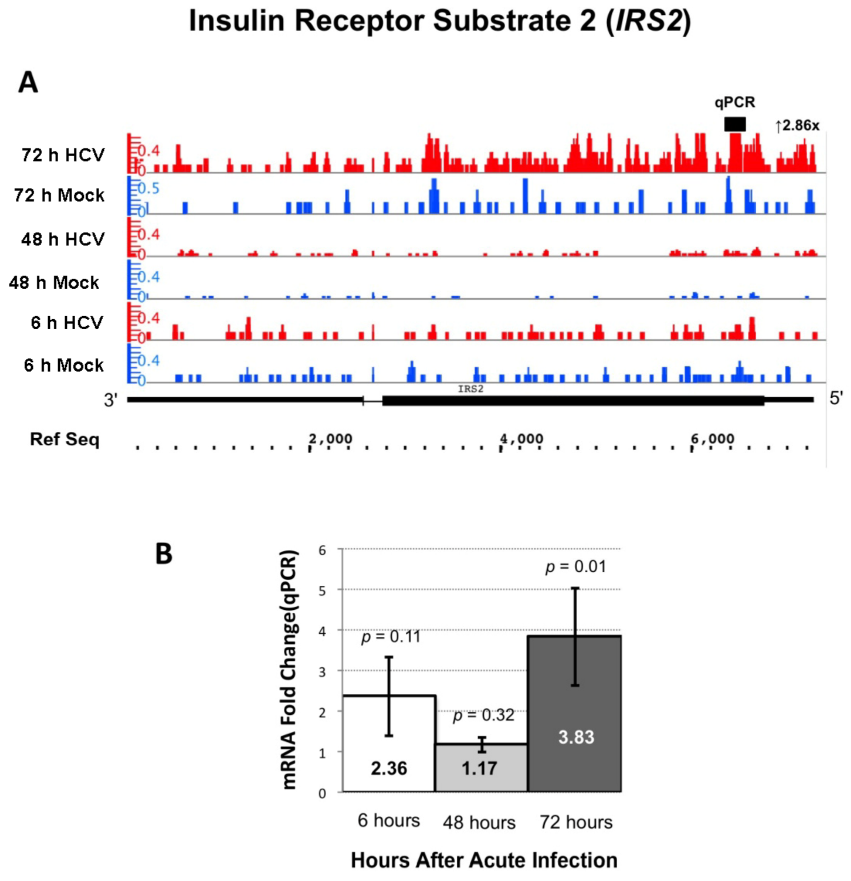

Figure S5. (**A**) Verification of *FUT1* and *KLHDC7B* siRNA gene expression knock-down Huh 7.5 cell cultures (triplicate for each condition and time in six well plates) by qPCR analysis (see Experimental Procedures). The control (open bar) represents cells transfected with a scrambled control siRNA. The data are presented as mean ±SEM. (**B**) Cytotoxicity assays were done under conditions that mimicked the siRNA studies. Huh 7.5 cells were plated in 96-well plate, cultured for 24 hours and transfected with siRNA against FUT1, KLHDC7B or control siRNA. Cells were mock infected 24 hours after transfection. Cell supernatants were collected (72 hours after transfection) and assayed for cellular toxicity using a lactate dehydrogenase cytotoxicity assay kit, CytoTox-ONE (Promega, Madison, WI, USA) according to manufacturer's recommendations (Experimental Procedures).

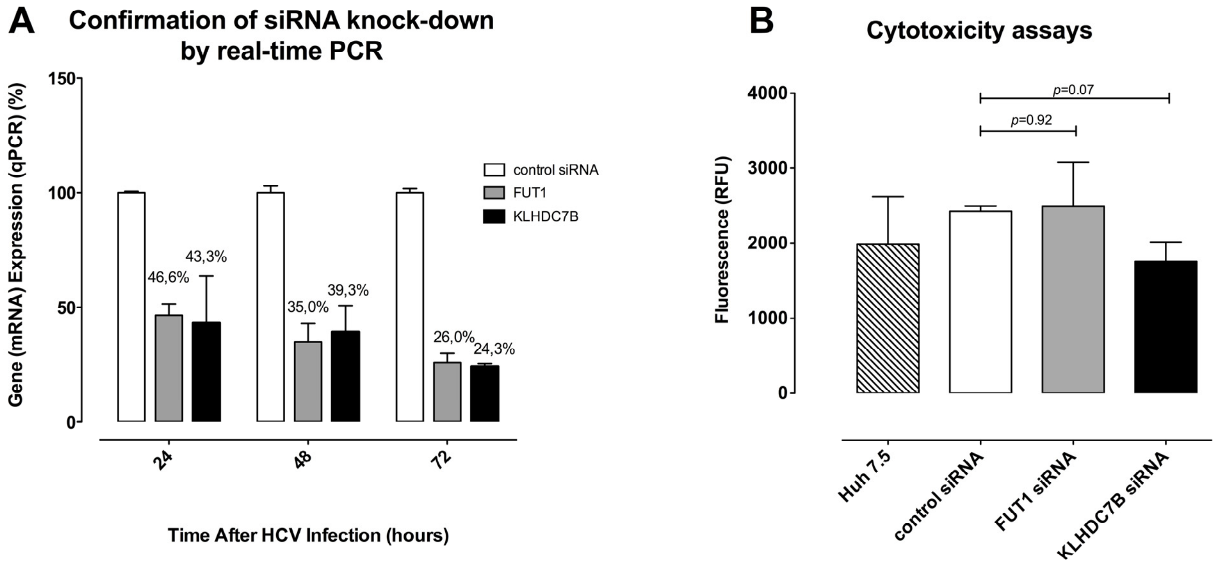

Supplement: Supplementary File 1: — PDF-Document (PDF, 1210 KB) [file viruses-04-00581-s001.pdf]
